# Variant restless legs syndrome masquerading as refractory shoulder pain: a case report and literature review

**DOI:** 10.3389/fmed.2025.1725747

**Published:** 2026-01-06

**Authors:** Peng Wang, Ling Yu, Liang Zhang, Jianyu Ren, Rongwei Qin, Guohong Luo, Siyu Chen

**Affiliations:** 1 Department of Internal Medicine, Nanchong Second People’s Hospital, Nanchong, China; 2 Department of Laboratory Medicine, Nanchong Second People’s Hospital, Nanchong, China; 3 Department of Geriatrics, Nanchong Second People’s Hospital, Nanchong, China; 4 Department of Psychiatry, Nanchong Second People’s Hospital, Nanchong, China

**Keywords:** shoulder pain, restless legs syndrome, restless arms syndrome, iron deficiency anemia, anxiety, depression, sleep disorders

## Abstract

Restless Legs Syndrome (RLS) is a common neurological disorder characterized by an irresistible urge to move the legs, often accompanied by uncomfortable sensations. These symptoms typically worsen during periods of rest or in the evening and are partially or temporarily relieved by movement. Variant RLS is diagnosed when these core symptoms extend to extra-leg regions—such as the arms, abdomen, or bladder—while still fulfilling the diagnostic criteria established by the International Restless Legs Syndrome Study Group. We report a case of an adult female patient with refractory right shoulder pain, ultimately diagnosed as variant RLS. Initially misdiagnosed as frozen shoulder, the correct diagnosis was confirmed through polysomnography, which revealed an elevated Periodic Limb Movement during Sleep index, and a supportive International RLS Rating Scale score. Following targeted treatment involving iron supplements for iron deficiency anemia and pramipexole for symptomatic relief, the patient’s shoulder pain and other associated symptoms improved markedly. This case expands the known clinical spectrum of variant RLS by illustrating a pain-dominant upper limb phenotype. Furthermore, we provide a summary analysis of previously reported cases of restless arms syndrome, highlighting the importance of including variant RLS in the differential diagnosis for patients presenting with unexplained shoulder or arm discomfort.

## 1 Introduction

Restless Legs Syndrome (RLS), also known as Willis-Ekbom Disease, is a common neurological sensorimotor disorder characterized by an irresistible urge to move the legs, accompanied by uncomfortable and often distressing sensations. These symptoms primarily emerge at rest or during the evening/night and are transiently relieved by movement. Epidemiological studies show that RLS prevalence in adults ranges from 1.9 to 4.6% in Europe and North America, higher than in Asian populations, and is more common in women than men (1). The clinical presentation of RLS is heterogeneous. When the core sensorimotor symptoms extend beyond the legs to involve other regions such as the arms, abdomen, or bladder, and still fulfill the essential diagnostic criteria established by the International Restless Legs Syndrome Study Group (2), the condition is termed variant RLS (3).

This report presents a unique case of an adult female patient whose primary manifestation was refractory right shoulder pain, ultimately diagnosed as a variant form of RLS. To the best of our knowledge, no previous literature has described refractory shoulder pain as the predominant feature of RLS. We hypothesize that this presentation may represent a specific and previously under-recognized phenotypic expression of restless arms syndrome, which falls under the broader spectrum of variant RLS. Such diagnostic ambiguity frequently leads to misdiagnosis and prolonged patient suffering. In addition to detailing this case, we provide a summary and analysis of 12 previously reported cases of restless arms syndrome. Our objective is to enhance clinical recognition of variant RLS, thereby facilitating accurate diagnosis and reducing the likelihood of diagnostic error.

## 2 Case report

A 51-year-old woman was admitted to our hospital with a two-month history of refractory right shoulder pain and poor sleep quality. The onset of her right shoulder pain was insidious, presenting as a persistent, dull ache localized to the right shoulder. A key feature was the diurnal variation, with symptoms exacerbating significantly at night. The nocturnal pain was severe and accompanied by arm discomfort, leading to severely disrupted sleep characterized by difficulty initiating sleep, frequent awakenings, and a total sleep duration of only approximately 3 h per night. Furthermore, she exhibited increased nocturnal motor activity, preferring to walk continuously. Rest, particularly lying down, aggravated the shoulder pain.

Prior to this admission, she had been diagnosed with frozen shoulder at a local clinic and treated with non-steroidal anti-inflammatory drugs and traditional Chinese medicine, which provided no significant relief. Over the following month, she developed restlessness, distractibility, and significant anxiety, primarily fueled by concerns about the intractable nature of her condition. As the right shoulder pain progressively intensified, she received a subsequent diagnosis of anxiety and depressive disorder at another facility. Treatment with escitalopram was initiated but yielded no clinical improvement.

In June 2025, she was subsequently admitted to our hospital for further management. Her past medical history was significant for hypertension of 20 years’ duration. Mild anemia was identified during a health check-up 2 years prior. The patient had no other chronic diseases, substance use history, or significant family history. She was an introverted farmer with local residency and primary education. Her gynecological history was notable for menorrhagia and two full-term deliveries, with menarche at age 14 and menopause at 50.

Physical examination on admission revealed pallor and conjunctival pallor. Vital signs were stable, with unremarkable cardiorespiratory and abdominal examinations. Mental status examination demonstrated intact orientation and cooperative behavior. Her thought process was logical, with no evidence of hallucinations or delusions. Speech was pressured but prosodic. She exhibited marked anxiety with comorbid depressive symptoms, reduced volition, and preserved insight. Notably, she displayed daytime social withdrawal and nocturnal restlessness, both exacerbated by her persistent pain.

Laboratory investigations revealed the following: White blood cell count 7.47 × 10^9^/L, Neutrophils 4.94 × 10^9^/L, Red blood cell count 3.49 × 10^12^/L, Hematocrit 24.6%, mean corpuscular hemoglobin (MCH) 22.4 pg, Hemoglobin 78 g/L, Platelets 290 × 10^9^/L. Iron studies showed serum ferritin <15 μg/L and transferrin saturation <15%. The Visual Analogue Scale pain score was 8/10. Hamilton Anxiety Scale = 21; Hamilton Depression Scale = 29; Montreal Cognitive Assessment = 8. Shoulder MRI was performed and revealed no structural abnormalities or nerve root compression. Nerve conduction studies of the upper limbs showed no evidence of peripheral neuropathy.

Initial management, including eszopiclone 3 mg nocte, duloxetine 30 mg daily, and acupuncture/physical therapy, provided minimal symptomatic improvement. A subsequent bone marrow aspiration demonstrated hypercellularity with erythroid hyperplasia and features consistent with iron deficiency, confirming Iron Deficiency Anemia. Treatment with Iron Dextran 50 mg and Vitamin C 0.5 g orally three times daily were initiated. Due to persistent nocturnal shoulder pain and poor sleep, a multidisciplinary consultation (Neurology, Orthopedics, Sleep Medicine) was held. Polysomnography revealed total sleep time of 4.2 h, an apnea-hypopnea index of 8.2/h (mild OSA), and a periodic limb movement during sleep (PLMS) index of 18.5/h (abnormal, >15/h), with a PLMS-associated arousal index of 7.2/h. No periodic upper limb movements were recorded, though the patient’s predominant complaint was shoulder pain. Based on the International Classification of Sleep Disorders, Third Edition (2014) and the diagnostic criteria established by the International Restless Legs Syndrome Study Group (2012), a final diagnosis of variant RLS was established. The treatment regimen was modified to include pramipexole (0.125 mg nocte, titrated to 0.25 mg nocte after 3 days), clonazepam 1 mg nocte and continuation of duloxetine 30 mg BID.

**Figure 1:**
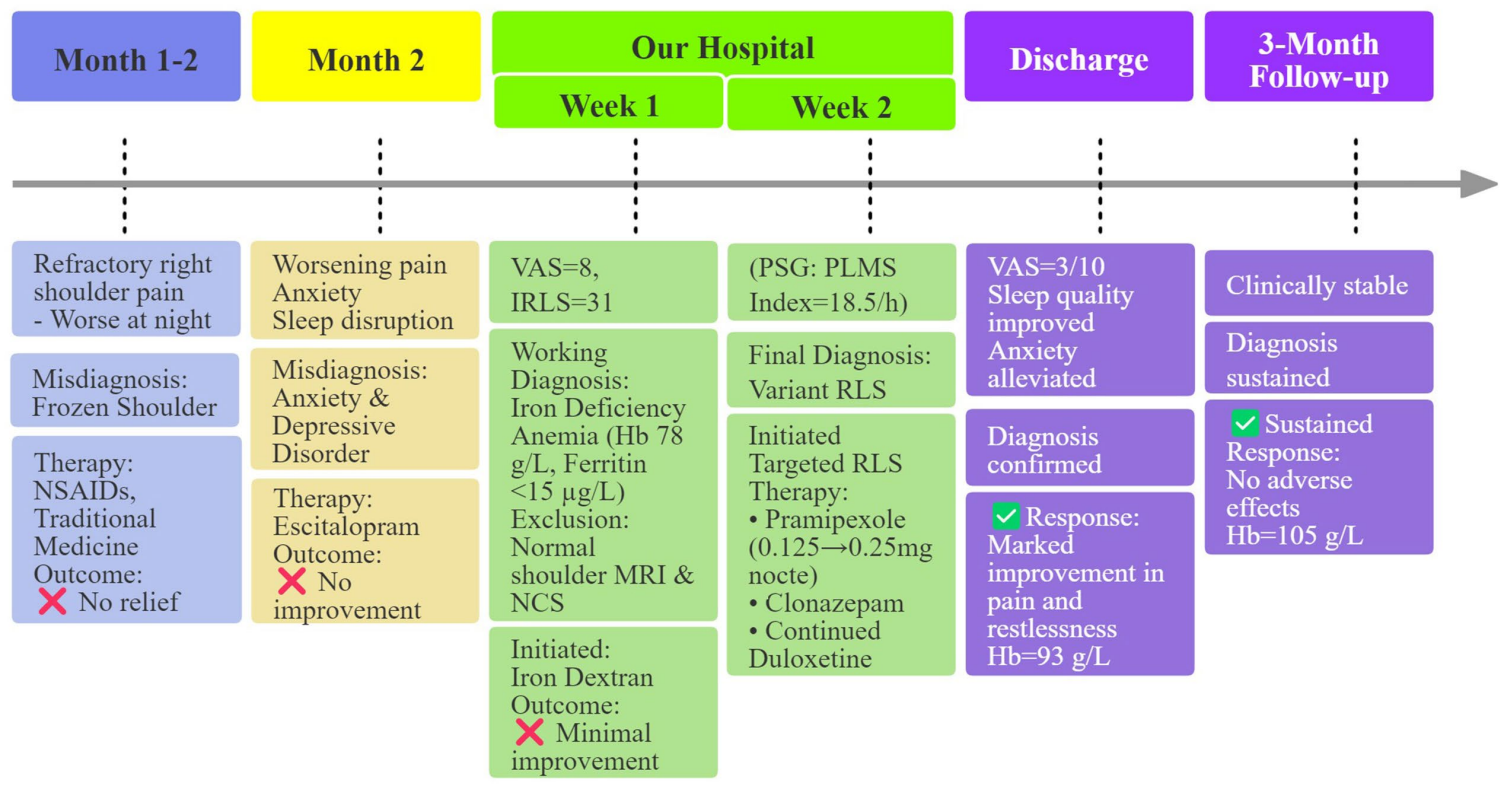
Flowchart of patient diagnosis and treatment this flowchart delineates the patient’s diagnostic odyssey and therapeutic response. Key milestones are highlighted, demonstrating the transition from initial misdiagnoses to the definitive diagnosis of variant RLS following a multidisciplinary evaluation. VAS, Visual Analogue Scale; IRLS, International Restless Legs Syndrome Rating Scale; NSAIDs, non-steroidal anti-inflammatory drugs; NCS, nerve conduction studies; PSG, Polysomnography; PLMS, Periodic Limb Movements during Sleep; RLS, Restless Legs Syndrome; Hb, Hemoglobin.

Following this targeted intervention, the patient’s right shoulder pain and sleep quality improved significantly, with resolution of nocturnal restlessness. Duloxetine was continued due to persistent anxiety symptoms and was recommended by the psychiatry consultation team. No exacerbation of RLS symptoms was observed after initiating pramipexole. While hemoglobin levels began to rise after iron initiation, the patient’s shoulder pain and sleep disturbance showed minimal improvement. In contrast, within 3 days of starting pramipexole, both nocturnal pain and restlessness markedly improved. At discharge, her anxiety had alleviated, mood was stable, VAS score decreased to 3/10 and Hemoglobin increased to 93 g/L and further to 105 g/L at the three-month follow-up, at which time no augmentation or significant adverse effects were reported. A timeline summarizing the patient’s diagnosis and treatment course is presented in Figure 1.

## 3 Discussion

RLS is a common neurological sensorimotor disorder primarily affecting the lower limbs. Beyond this classical presentation, various clinical variants have been recognized, including restless arms syndrome, restless abdomen syndrome, and restless genital syndrome (3). Since its initial formal description in 1976 (4), relatively few cases of restless arms syndrome have been documented (5). Consequently, clinical awareness of these RLS variants remains inadequate, frequently leading to misdiagnosis or delayed diagnosis (6). An earlier case report described an elderly woman diagnosed with restless arms syndrome presenting as “abnormal sensations in both shoulders accompanied by insomnia,” whose symptoms resolved following pramipexole treatment (7). Our case was characterized by refractory shoulder pain as the dominant symptom, accompanied by arm discomfort, suggesting a possible pain-predominant phenotypic variant of restless arms syndrome. To our knowledge, this is the first reported case of a variant RLS manifesting primarily as refractory shoulder pain.

To better delineate the clinical spectrum of this entity, we conducted a systematic literature search in PubMed, Embase, and Web of Science from inception until September 2025, using the following key terms and their combinations: “restless arms syndrome,” “upper limb RLS,” “variant RLS.” Inclusion Criteria: Patients diagnosed with restless legs syndrome (RLS) or variant RLS where symptoms primarily involved the upper limbs (arms, shoulders, or hands). The exclusion criteria were: (a) cases with symptoms exclusively in the lower limbs; (b) non-English publications; and (c) cases with insufficient clinical data to confirm the diagnosis or phenotype. Twelve cases of restless arms syndrome met the inclusion criteria (Table 1). Existing literature indicates that restless arms syndrome typically presents with paroxysmal sensory disturbances—such as pruritus, tingling, or burning—or involuntary movements, including twitching, myoclonus, or tremor, and appears to exhibit a male predilection. In striking contrast, our case involves a female patient with refractory, localized shoulder pain, thereby expanding the known phenotypic spectrum of restless arms syndrome. This fundamental shift in presentation—from transient sensory phenomena to persistent nociceptive pain—suggests the involvement of distinct neurophysiological mechanisms, potentially reflecting aberrant central pain processing coexisting with the classic dopaminergic dysfunction of RLS.

**Table 1:** Summary of reported cases with restless arms syndrome.

No.	Sex	Age	Primary clinical presentation	Leg symptoms	Precipitating factors	Treatment	Reference
1	M	78	Arms, twitching and wiggling sensations	Yes	N/A	Gabapentin	(18)
2	M	66	Back, shoulders and arms, pain	No	N/A	Levodopa/Benserazide	(19)
3	F	48	Discomfort, Arms	No	Olanzapine	N/A	(20)
4	F	33	Itching, Arms	No	N/A	N/A	(21)
5	F	67	bilateral shoulders and arms, itching and creeping sensation	Yes	Duloxetine	Pramipexole	(22)
6	M	40	movements of repeated extension, Small finger	No	N/A	Pramipexole	(23)
7	M	33	Reduced sensation, arms and legs	Yes	N/A	N/A	(24)
8	M	24	Tingling and pricking, Arms	No	Olanzapine	N/A	(25)
9	M	39	Burning and itching, Arms	Yes	N/A	Pramipexole	(26)
10	M	23	Aimless movements, Arms	No	Spinal cord injury	N/A	(4)
11	M	73	Myoclonic-like jerks, Arms	No	N/A	N/A	(5)
12	M	59	Tremor and itching, Arms	Yes	Surgery	Pramipexole	(27)

N/A, not available or not applicable.

Accurate identification of variant RLS, particularly when initial or predominant symptoms manifest outside the lower limbs, is essential to prevent missed and misdiagnosis. This was exemplified in our case of a middle-aged woman whose refractory right shoulder pain was initially misdiagnosed as frozen shoulder and later as an anxiety-depressive disorder, with poor response to corresponding treatments. The definitive diagnosis of RLS was ultimately established through a multidisciplinary diagnostic workup at our institution, which included critical findings from polysomnography and bone marrow aspiration. Following the initiation of combination therapy with iron, pramipexole, and clonazepam, the patient’s nocturnal ambulation and sleep disturbances demonstrated marked improvement. Crucially, her refractory shoulder pain also resolved substantially. The simultaneous resolution of classic RLS symptoms and shoulder pain provides strong clinical evidence for a direct association, highlighting the importance of recognizing this pain-dominant phenotype in variant RLS.

The International Restless Legs Syndrome Rating Scale (IRLS) is a well-validated tool for quantifying RLS severity (8). In this case, the patient’s IRLS score of 31 (Table 2) indicated very severe disease. Although the patient did not report spontaneous leg discomfort, the IRLS questionnaire indicated mild leg symptoms (score 3/4 for leg or arm discomfort). Clinicians should be aware that focal pain, particularly when refractory to conventional therapies, may have an underlying neurological etiology rather than a primary musculoskeletal origin. The patient’s poor response to initial analgesic and physical therapies can be explained by the distinct pathophysiology of RLS, which involves central dopaminergic dysfunction (9). Dopamine plays a critical role not only in motor regulation but also in central pain modulation (10), and its deficiency may directly contribute to the nociceptive symptoms observed in this case. Although the temporal association between RLS-directed therapy and symptom resolution is suggestive, this remains a single case observation, and alternative explanations such as placebo effect or spontaneous remission cannot be entirely excluded.

**Table 2:** International restless legs syndrome study group rating scale.

No.	Question	Very severe (4 points)	Severe (3 points)	Moderate (2 points)	Mild (1 point)	None (0 points)	Score
1	Overall, how would you rate the RLS discomfort in your legs or arms?	Very severe	Severe	Moderate	Mild	None	3
2	How would you rate the need to move around because of your RLS symptoms?	Very severe	Severe	Moderate	Mild	None	3
3	How much relief of your RLS arm or leg discomfort do you get from moving around?	No relief / Mild relief	Moderate relief	Either complete or almost complete relief	Complete relief	No symptoms to relieve	2
4	How severe is your sleep disturbance due to your RLS symptoms?	Very severe	Severe	Moderate	Mild	None	3
5	How severe is your tiredness or sleepiness during the day due to your RLS symptoms?	Very severe	Severe	Moderate	Mild	None	3
6	How severe is your RLS condition as a whole?	Very severe	Severe	Moderate	Mild	None	3
7	How often do you get RLS symptoms?	Very often (6–7 days a week)	Often (4–5 days a week)	Sometimes (2–3 days a week)	Occasionally (≤1 day a week)	Never	4
8	When you have RLS symptoms, how severe are they on average?	Very severe (≥8 h per 24 h)	Severe (3–8 h per 24 h)	Moderate (1–3 h per 24 h)	Mild (<1 h per 24 h)	None	4
9	How severe is the impact of your RLS symptoms on your ability to carry out your daily affairs?	Very severe	Severe	Moderate	Mild	None	3
10	How severe is your mood disturbance due to your RLS symptoms?	Very severe	Severe	Moderate	Mild	None	3
	Total						31

Total Score Range: 0–40, 0: None, 1–10: Mild RLS, 11–20: (2) Moderate RLS, 21–30: Severe RLS, 31–40: Very severe RLS. Shoulder pain is also considered a component of RLS symptoms in this table.

RLS is clinically categorized into primary (idiopathic) and secondary forms. While its exact pathophysiology is not fully understood, current evidence points to a complex interplay of dopaminergic dysfunction, central iron deficiency, and genetic susceptibility (11). Dysregulation of other central nervous system neurotransmitters may also contribute (11). Additionally, several predisposing factors are well-established, including renal impairment, pregnancy, and iron deficiency anemia (12), all of which can trigger or exacerbate RLS symptoms. In the present case, the patient had iron deficiency anemia secondary to menorrhagia—established predisposing factors for RLS. Previous studies (13)report that 25–35% of patients with iron deficiency anemia develop RLS, with symptom occurrence being independent of anemia severity. This correlation can be explained by the crucial role of iron as a cofactor for tyrosine hydroxylase, the rate-limiting enzyme in dopamine synthesis; insufficient iron levels thus directly impair dopaminergic neurotransmission (14). In this patient, iron deficiency is considered the principal trigger, as RLS symptoms emerged only recently despite the long-standing anemia of 2 years’ duration. Due to severe iron deficiency and the unavailability of intravenous iron preparations, iron dextran is administered orally at a dose of 50 mg three times a day. The recent onset of RLS despite chronic anemia may reflect individual variability in brain iron regulation or a threshold effect in dopaminergic dysfunction. We propose that the patient’s predominant right shoulder pain may stem from iron deficiency-induced impairment of the nigrostriatal dopaminergic pathway. This dysregulation could potentially affect adjacent cervical spinal cord circuits or higher-order sensorimotor integration centers, ultimately generating restless legs syndrome-like symptoms in the upper limb, with a notable predilection for the shoulder region. Nevertheless, the precise pathophysiological mechanisms responsible for the phenotypic diversity in variant RLS require further elucidation. Although the patient showed no parkinsonian features and responded well to low-dose pramipexole, long-term neurological follow-up is recommended. A DAT scan was not performed as there were no clinical signs suggestive of parkinsonism, but could be considered in future if symptoms evolve.

Furthermore, the chronic physical discomfort associated with RLS frequently leads to secondary anxiety and depressive disorders. In clinical practice, there is a risk of overemphasizing these psychiatric symptoms while overlooking the underlying somatic manifestations of RLS (15). Therefore, it is critical to distinguish whether affective symptoms are the cause or the consequence of RLS, and to recognize the substantial psychiatric burden often attributable to the disorder itself (16). Previous studies (17)have reported that up to 61% of RLS patients experience pain, which in turn predisposes them to daytime drowsiness and fatigue. More than one-third of patients describe their symptoms in affectively laden terms such as “annoying,” “exhausting,” or “unbearable.” In the present case, the patient had previously received antidepressant and anxiolytic therapy both before and during admission, yet her shoulder pain and sleep disruption persisted. In contrast, her mood and anxiety improved rapidly following targeted RLS treatment, supporting the conclusion that her psychiatric symptoms were secondary to the underlying sensorimotor disorder. Thus, in patients presenting with refractory somatic pain, sleep disturbances, and affective symptoms, clinicians should maintain a high index of suspicion for a potential underlying diagnosis of RLS.

## 4 Limitations

This study has several limitations. First, it is a single case report, which limits the generalizability of our findings. Second, the potential confounding effects of multiple medications, although carefully considered, cannot be entirely ruled out. Finally, the MoCA score of 8 was assessed during severe sleep deprivation and anxiety, likely not reflecting baseline cognitive function, which may affect the reliability of self-reported symptoms.

## 5 Conclusion

This case underscores that variant RLS can present as isolated, refractory shoulder pain, thereby broadening the known clinical spectrum of the disorder. In patients presenting with nocturnal upper-limb pain unresponsive to conventional musculoskeletal treatments, clinicians should inquire about core RLS features—such as an urge to move, rest-induced symptom onset, and evening exacerbation—to include variant RLS in the differential diagnosis and prevent misdiagnosis.

## Data Availability

The original contributions presented in the study are included in the article/supplementary material, further inquiries can be directed to the corresponding author.
